# GLP-1-Induced AMPK Activation Inhibits PARP-1 and Promotes LXR-Mediated ABCA1 Expression to Protect Pancreatic β-Cells Against Cholesterol-Induced Toxicity Through Cholesterol Efflux

**DOI:** 10.3389/fcell.2021.646113

**Published:** 2021-07-07

**Authors:** Rao Li, Xulong Sun, Pengzhou Li, Weizheng Li, Lei Zhao, Liyong Zhu, Shaihong Zhu

**Affiliations:** ^1^Department of Gastrointestinal Surgery, The Third Xiangya Hospital, Central South University, Changsha, China; ^2^Department of General Surgery, First Affiliated Hospital of University of South China, Hengyang, China

**Keywords:** T2DM (type 2 diabetes), GLP-1, β-cell, PARP-1, ABCA1

## Abstract

T2DM (Type 2 diabetes) is a complex, chronic disease characterized as insulin resistance and islet β-cell dysfunction. Bariatric surgeries such as Roux-en-Y gastric bypass (RYGB) surgery and laparoscopic sleeve gastrectomy (LSG) have become part of a critical treatment regimen in the treatment of obesity and T2DM. Moreover, GLP-1 increase following bariatric surgery has been regarded as a significant event in bariatric surgery-induced remission of T2DM. In this study, a high concentration cholesterol-induced lipotoxicity was observed in INS-1 cells, including inhibited cell viability and insulin secretion. Enhanced cell apoptosis and inhibited cholesterol efflux from INS-1 cells; meanwhile, ABCA1 protein level was decreased by cholesterol stimulation. Cholesterol-induced toxicity and ABCA1 downregulation were attenuated by GLP-1 agonist EX-4. GLP-1 induced AMPK phosphorylation during the protection against cholesterol-induced toxicity. Under cholesterol stimulation, GLP-1-induced AMPK activation inhibited PARP-1 activity, therefore attenuating cholesterol-induced toxicity in INS-1 cells. In INS-1 cells, PARP-1 directly interacted with LXR, leading to the poly(ADP-ribosyl)ation of LXRα and downregulation of LXR-mediated ABCA1 expression. In the STZ-induced T2DM model in rats, RYGB surgery or EX-4 treatment improved the glucose metabolism and lipid metabolism in rats through GLP-1 inhibition of PARP-1 activity. In conclusion, GLP-1 inhibits PARP-1 to protect islet β cell function against cholesterol-induced toxicity *in vitro* and *in vivo* through enhancing cholesterol efflux. GLP-1-induced AMPK and LXR-mediated ABCA1 expression are involved in GLP-1 protective effects.

## Introduction

Type 2 diabetes mellitus (T2DM) is a complex, chronic disease that is characterized by insulin resistance and islet β-cell dysfunction (Thorens, 2013). Glucagon-like peptide-1 (GLP-1) has been reported to improve T2DM through the stimulation of insulin-secreting pancreatic β-cells (Kreymann et al., 1987; Buchwald et al., 2004), and the inhibition of glucagon-secreting pancreatic α-cells (Holst, 2004), and decreasing gastrointestinal motility (Meier et al., 2003) and body weight (Larsen et al., 2001). Coupled with its specific receptor GLP-1 receptor (GLP-1R), GLP-1/GLP-1R signaling protects pancreatic β-cells through AMPK/mTOR, PI3K, and Bax (Huang et al., 2017).

Cholesterol metabolism disorders are an important mechanism in islet dysfunction (Brunham et al., 2008). The maintenance of cholesterol homeostasis would improve the function of pancreatic β cells. ATP-binding cassette transporter A1 (ABCA1) has been considered the primary regulator of cholesterol efflux (Westerterp et al., 2013). The liver X receptor (LXR) could transcriptionally and post-transcriptionally regulate ABCA1 expression. Moreover, the direct interaction of LXR with ABCA1 promotes the formation of HDL (Singaraja et al., 2003; Tall, 2003). In mice, the uptake of low-density lipoprotein (LDL) receptor (LDLr)-mediated cholesterol into pancreatic β-cells strongly affects islet cholesterol levels and β-cell function. Contrastingly, ABCA1-mediated cholesterol efflux could simultaneously compensate for hypercholesterolemia to balance islet cholesterol levels *in vivo* (Kruit et al., 2010). However, the impact of the LXR-mediated ABCA1 expression upon the regulation of blood glucose homeostasis is unclear as of yet.

Poly ADP-ribose polymerase (PARP) gene plays a critical role in inducing the damage of diabetic islet cells. In Streptozotocin (STZ)-induced diabetic mouse model, DNA damage and PARP activation in islet cells cause islet cell apoptosis, islet destruction, and increased blood glucose. In contrast, in PARP^–/–^ mice, normal blood glucose and islet structure have been observed, indicating that the deletion of PARP can completely protect STZ-induced diabetes (Pieper et al., 1999). AMPK downstream of GLP-1 can rapidly inhibit PARP activity and reduce its ability to regulate downstream genes (Gongol et al., 2013; Shang et al., 2016). Besides, PARP-1 regulates cholesterol efflux in macrophages by inhibiting LXR-mediated ABCA1 expression (Shrestha et al., 2016). However, the effects of PARP-1 on LXR-mediated ABCA1 expression and cholesterol efflux from β-cells remain unknown. It is, therefore, speculated that in islet cells, GLP-1 inhibits PARP-1 after the activation of AMPK, and the down-regulation of PARP activity increases LXR transcriptional activity. As a result, ABCA1 expression increases, promotes cholesterol outflow and reduces cholesterol-induced cytotoxicity.

Conventional therapies, including anti-diabetic drug therapy, only slow down the onset of TD2M progression and do not remotely heal the disease. Comparatively, a series of bariatric surgeries and mainly Roux-en-Y gastric bypass (RYGB) surgery and LSG, have become an essential method in the treatment of obesity and T2DM in recent years, especially for cases where the curative effect of the standardized medical treatment is not satisfactory (Dixon et al., 2008; Bradley et al., 2012; Reis et al., 2012). A study on 35 T2DM patients having undergone bariatric surgery (23 RYGB and 12 SLG) indicated that bariatric surgery increased the insulin secretion rates and concentrations, slightly improved β-cell and rate sensitivity, and enhanced glucagon and GLP-1 response (Nannipieri et al., 2013). Following RYGB, the GLP-1 concentrations in postprandial plasma were markedly increased (Laferrere et al., 2007). Since GLP-1 is an incretin hormone playing an anti-diabetic role, GLP-1 increase after bariatric surgery has also been regarded as a central event in RYGB-induced remission of T2DM (Rhee et al., 2012).

To validate the above-described speculation, the effects of GLP-1 on ABCA1 expression and cholesterol-induced toxicity in islet β INS-1 cells and the involvement of AMPK were examined. Next, the effects of GLP-1-induced AMPK activation on PARP-1 activity and ABCA1 expression were examined. INS-1 cells were then treated with AMPK agonist-treated and/or PARP-1 under cholesterol stimulation; the dynamic effects were subsequently monitored. The interaction between PARP-1 and LXR was verified, and the effects of PARP-1 on LXR-mediated ABCA1 expression were determined. To further validate the *in vitro* findings, an STZ-induced T2DM model in rats was established, and RYGB surgery was performed on T2DM rats, or T2DM rats were alternatively treated with GLP-1R agonist EX-4 and examined glucose metabolism- and lipid metabolism-related indicators were examined. In summary, a novel mechanism was attempted through which increased GLP-1 after bariatric surgery increases LXR-mediated ABCA1 expression through the inhibition of PARP-1 activity, therefore protecting INS-1 cells from cholesterol-induced toxicity.

## Materials and Methods

### Cell Line and Cell Treatment

INS-1 cells were procured from the National Infrastructure of Cell Line Resource (Beijing, China) and cultured in RPMI-1640 (Invitrogen, Carlsbad, CA, United States) added with L-glutamine, FBS (10%; Invitrogen), HEPES (10 mmol/L; Sigma, St. Louis, MO, United States), sodium pyruvate (1 mmol/L; Invitrogen), and β-mercaptoethanol (50 μmol/L; Sigma-Aldrich, ıSt. Louis, MI, United States) at 37°C in 5% CO_2_.

For high-fat induction, cholesterol (Santa Cruz Biotechnology; Dallas, TX, United States) was dissolved in chloroform and diluted to 1, 2.5, and 5 and 10 mmol/L with a culture medium for 24 h (Kong et al., 2017); For GLP-1 treatment, cells were treated with GLP-1 agonist at a concentration of 10 nM exendin-4 (Ex-4) for 24 h (Zhu et al., 2014); For AMPK activation or inhibition, according to the aforementioned procedure (Zhang et al., 2009; Shang et al., 2016), cells were pre-treated with 0.5 mM Acadesine (AICAR, an AMPK agonist) or 10 μM Compound C (an AMPK antagonist) for 4 h and subsequently co-treated with 10 nM Ex-4 for 24 h, respectively. For PARP-1 inhibition, INS-1 cells were treated with 5 mM 3-aminobenzaminde (3-AB) (Sigma) for 24 h. For LXR receptor activation, INS-1 cells were treated with 5 μM T0901317 for 4 h (Shrestha et al., 2016).

### Cell Transfection

PARP-1 knockdown was generated in target cells through the transfection of si-PARP-1 synthesized by GenePharma (Shanghai, China). PARP-1 overexpression was generated in target cells by the transfection of PARP-1-pcDNA3.1 overexpression vector by (GenePharma). The transfection into target cells was performed with lipofectamine 3,000 (Invitrogen). Twenty-four hours later, the transfected cells were harvested for unrelated experiments.

### Cell Viability Evaluation

The cell viability was examined using 3-[4,5-dimethylthiazol-2-yl]-2,5-diphenyl tetrazolium bromide (MTT) in line with the aforementioned methods (Liu H. et al., 2018). MTT was taken up by cells through the plasma membrane potential and then reduced to formazan by intracellular NAD(P)H-oxidoreductases, and the formazan was dissolved by DMSO after discarding the supernatant. OD values were measured at 490 nm at 48 h, and the viability was calculated, taking the non-treated cell (control) viability as 100%.

### Cell Apoptosis Evaluation

Cell apoptosis was determined through Flow cytometry. The cells were digested by trypsin and collected. Following resuspension in 500 μl binding buffer, the cells were added with 5 μl Annexin V-FITC and 5 μl Propidium Iodide (PI) (KeyGene, Nanjing, China) at room temperature in the dark for 15 min. The cells were subsequently tested by flow cytometry.

### Immunoblotting

The total protein from cells or tissues were extracted by RIPA lysis buffer (Beyotime, Shanghai, China). 50 μg protein were separated by 10% SDS-PAGE, and transferred onto polyvinylidene fluoride (PVDF) membranes. Non-specific bindings were blocked by incubation with 5% non-fat dry milk in Tris-buffered saline Tween (TBST) for 2 h. Then, membranes were incubated with the appropriate primary antibodies at 4°C overnight. The primary antibodies used are as follows: anti-ABCA1 (1:500, ab18180, Abcam, Cambridge, United Kingdom), anti-PARP-1 (1:1000, 13371-1-AP, Proteintech, Wuhan, China), anti-p-PARP-1 (1:1000, MBS9206900, MyBioSource, San Diego, CA, United States), anti-LXRα (1:500, ab176323, Abcam), anti-AMPK (1:1000, 10929-2-AP, Proteintech), anti-p-AMPK (1:1000, ab133448, Abcam). Then, the membranes were further incubated with secondary antibodies (goat-anti rabbit or anti mouse antibodies, Beyotime, Shanghai, China) for 2 h at room temperature. The immunoreactive proteins were visualized and examined using an enhanced chemiluminescence reagent (ECL; BeyoECL Star Kit, Beyotime) and an automatic chemiluminescence imaging system (Tannon-4200, China).

### Polymerase Chain Reaction (PCR)-Based Analyses

Total RNA was extracted, processed, and examined for the expression of target genes in line with an aforementioned method (Liu Y. et al., 2018). The expression levels of target genes were detected by SYBR green PCR Master Mix (Qiagen, Hilden, Germany), taking GAPDH as an endogenous control. The data were processed using a 2^–ΔΔCT^ method.

### Measurements of Cellular Cholesterol

INS-1 cells were received different treatments for 24 h. The cellular contents of cholesterol in INS-1 cells were measured using Tissue and cell TC enzymatic assay kit (Applygen, Beijing, China) according to the manufacturer’s protocol. The cellular protein levels were determined by a bicinchoninic acid assay (Beyotime). The cellular cholesterol levels were presented mmol cholesterol/mg protein.

### Measurements of Insulin Secretion

INS-1 cells were received different treatments for 24 h. After undergoing two washes with PBS, the cells were serum-deprived for 1 h in Krebs–Ringer bicarbonate HEPES (KRBH) buffer including 1% bovine serum albumin (BSA), followed by KRBH buffer containing 1% BSA and 11.1 mM glucose for 0.5 h. The secreted insulin was measured in the supernatants using a rat insulin ELISA kit (Elabscience Biotechnology Co., Ltd., Wuhan, China). After the lysis of the cells with RIPA lysis buffer, the cellular protein levels were measured using a bicinchoninic acid assay (Beyotime). Insulin levels were presented as ng insulin/μg protein.

### Determination of Adipogenic Induction

To identify the lipid droplets in cells following treatment and/or after transfection, INS-1 cells were fixed in paraformaldehyde (4%), stained with oil red O (0.5%; Santa Cruz, Dallas, TX, United States), and representative images were captured under an inverted light microscope. For BODIPY staining, fixed cells were stained by a BODIPY solution (1 μg/ml), and representative images were captured under a fluorescence microscope.

### Cholesterol Efflux From INS-1 Cells

The BODIPY-cholesterol assay was performed to monitor the cholesterol efflux from INS-1 cells as per the aforementioned methods (Shrestha et al., 2016). Cells were plated in black 96-well plates at a density of 2 × 10^4^ cells/well and cultured for 24 h. The cells were subsequently received different treatments for 24 h and were cultured in a serum-free medium containing a labeling media, which consisted of BODIPY cholesterol (0.025 mM) (BODIPY-Cholesterol, Avanti Polar Lipids, Alabaster, AL, United States), MCD (10 mM, sigma), HEPES (Sigma), and egg phosphatidylcholine (0.1 mM) (Avanti Polar Lipids) (Sankaranarayanan et al., 2011) for 1 h. The cells were washed with RPMI-1640 and received different treatments for 24 h. The culture medium was collected for fluorescence intensity determination. The cells were subsequently dissolved within 1N NaOH for 4 h. After centrifuged at 10,000 × *g* for 5 min the lysate supernatant was collected or fluorescence intensity determination. The fluorescence intensity representing the cholesterol was examined using a microplate reader at an excitation wavelength of 482 nm and an emission wavelength of 515 nm. The percentage of the cholesterol efflux was calculated as cholesterol efflux intensity/(intracellular cholesterol + cholesterol efflux) × 100%. The relative cholesterol efflux was normalized to control group.

### Co-immunoprecipitation (Co-IP) Assay

The sequence encoding LXR and PARP-1 were cloned into the pcDNA3.1/Flag or pcDNA3.1/His vector, named Flag-LXR and His-PARP-1, respectively. These vectors were subsequently co-transfected into INS-1 cells. Empty vectors were co-transfected into target cells to act as controls. Thirty-six hours after transfection, the cells were harvested, and the proteins were extracted. Flag monoclonal antibodies were used for IP testing, followed by immunoblotting using anti-Flag (ab49763; Abcam) and anti-His (ab5000; Abcam) antibodies. To exclude the effect of DNase and RNase, the cell lysates were treated with 5 mg/ml Dnase and Rnase, respectively.

### Poly (ADP-Ribosyl)ation Assay

To examine poly (ADP-ribosyl)ation (also known as PARylation, is a special case of ADP-ribosylation), recombinant full-length human GLP-1 (100 ng; Abcam) were incubated with recombinant human PARP-1 (40 ng; 31238; Active Motif, Carlsbad, CA, United States) with the pre-treatment of AMPK activator AICAR or AMPK inhibitor Compound C under cholesterol treatment, or recombinant full-length human LXRα (100 ng; Protein One, Rockville, MD, United States) was incubated with recombinant human PARP-1 (40 ng; 31238; Active Motif) in the reaction buffer as described previously (Shrestha et al., 2016) for 30 min. The reaction was terminated as described, and the poly (ADP-ribosyl) ation was visualized by immunoblotting with an anti-PAR1 antibody (Shrestha et al., 2016).

### Diabetes Mellitus Rat Models and Roux-en-Y Gastric Bypass (RYGB) Surgery

Six-week-old male SD rats (*n* = 50) were procured from the SLAC Laboratory Animal Co., Ltd. (Shanghai, China) and randomly assigned into five groups: the non-treatment control group, the high-fat diet (HFD)/streptozotocin (STZ)-induced T2DM group, the STZ-induced T2DM plus sham surgery group, the STZ-induced T2DM plus RYGB surgery group, and the STZ-induced T2DM plus EX-4 treatment group. To establish the T2DM model, after 8 weeks of HFD (10% lard, 20% sucrose, 2.5% cholesterol, 1% bile salt, and 66.5% regular diet), rats received the intraperitoneal (i.p.) injection of 35 mg/kg STZ (Sigma) after overnight fasting. Two weeks later, the blood glucose was measured three times randomly, and the average value was more than 16.7 mmol/L. After the successful establishment of the model, rats in the T2DM plus EX-4 treatment group received a subcutaneous injection of 5 μg/kg EX-4 twice daily for 8 weeks. Rats in the non-treatment control group were fed a regular diet and injected with an equal volume of PBS in citrate buffer solution (pH 4.5). Rats in the T2DM plus RYGB surgery or sham surgery group were starved overnight and then intraperitoneally anesthetized with chloral hydrate (10%, 350 mg/kg; Sigma). The RYGB surgery and sham surgery were performed on rats following the aforementioned methods (Gatmaitan et al., 2010). The animal experiments were approved by the Ethics Committee of The Third Xiangya Hospital of Central South University.

### Blood and Pancreatic Tissue Sample Collection and Examination

For animal experiments, blood was collected from the rats’ inner canthus vein before and on weeks 1, 2, 4, and 8 after surgery or EX-4 injection. For pancreatic tissue sample collection, rats in different groups were sacrificed by euthanasia, and the pancreases were stored at −80°C or fixed in 10% formalin for pending further analysis. GLP-1 and insulin levels in rat blood samples were determined by an ELISA assay using GLP-1 (cat. no. RA20061; Bio-Swamp Life Science, Wuhan, China) and insulin (cat. no. RA20092; Bio-Swamp Life Science) kits following the manufacturer’s instructions. The levels of total cholesterol (TC), triglyceride (TG), low-density lipoprotein cholesterol (LDL-C), and high-density lipoprotein cholesterol (HDL-C) in serum were determined using an automatic biochemical analyzer (BS-450; Mindray, Nanshan, Shenzhen, China). TC and TG levels in the pancreatic tissues were determined using Tissue and cell TC enzymatic assay kit and Tissue and cell TG enzymatic assay kit (Applygen, Beijing, China).

### Hematoxylin and Eosin (H&E) and Immunohistochemical (IHC) Staining

Formalin-fixed and paraffin-embedded rats’ pancreatic tissue sections were deparaffinized in xylene and rehydrated using graded ethanol into PBS. Endogenous peroxidase was blocked with 0.3% hydrogen peroxide in methanol. The sections were stained with H&E pending histopathological analysis. For the protein content and distribution, the sections were applied for IHC staining. Sections were incubated with 5% normal rat serum followed by further incubation; firstly, with primary antibodies against ABCA1 (ab18180, Abcam) and GLP-1 (ab26278, Abcam) at 4°C overnight, and secondly, with a biotinylated secondary antibody, and thirdly, with an avidin-biotinylated peroxidase complex (Santa Cruz Biotechnology). The HE and IHC staining sections were observed and determined through a double-blind study by two authors. Cytoplasmic staining for ABCA1 or GLP-1 was considered positive. Five random visual fields from each section were selected and analyzed under 400× magnification. The positive area were calculated, which is showed in percentage (ratio of positive area to the whole visual field).

### Data Processing and Statistical Analysis

The data were analyzed with GraphPad software. The measurement data were expressed as mean ± standard deviation (SD). Among-group and intra-group data comparisons were performed with the ANOVA and Student’s *t*-tests. *P* < 0.05 indicated a statistically significant difference.

## Results

### GLP-1 Upregulates ABCA1 Expression to Inhibit Cholesterol Accumulation-Induced Toxicity in INS-1 Cells

**FIGURE 1:**
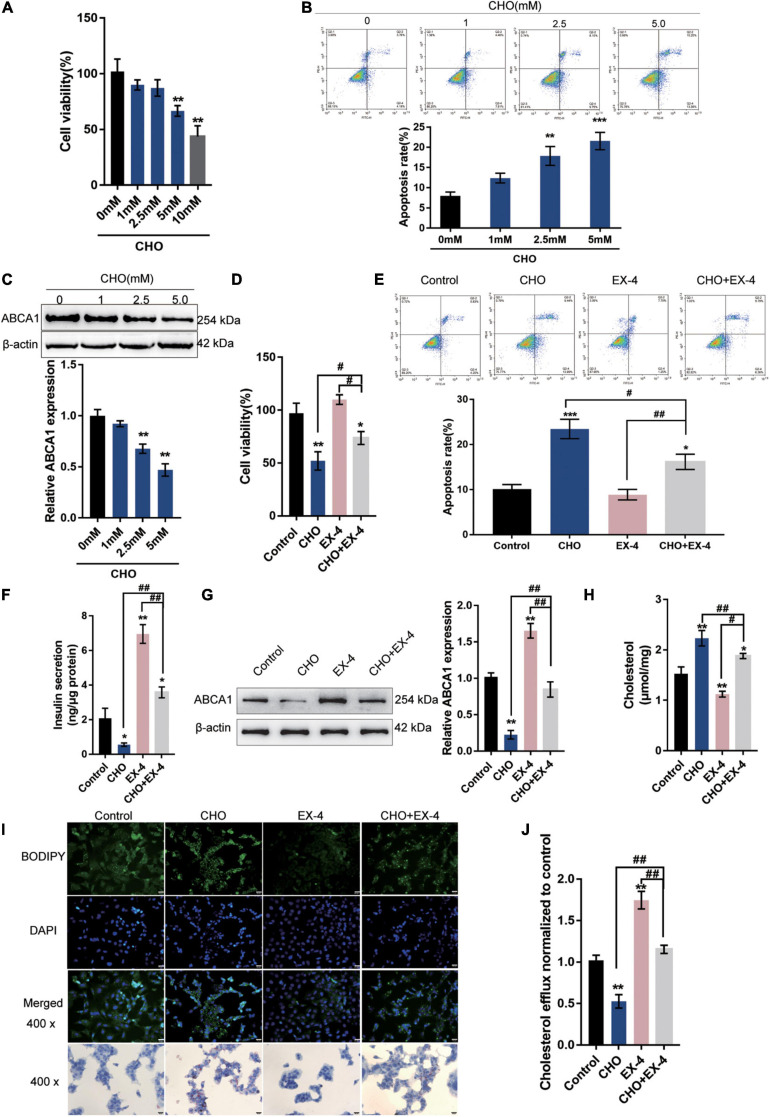
GLP-1 downregulates ABCA1 expression and cholesterol accumulation-induced toxicity in INS-1 cells. INS-1 cells were cultured in a medium containing a series of concentrations of cholesterol (0, 1, 2.5, 5, or 10 mM) for 24 h and examined for **(A)** cell viability by MTT assay; **(B)** cell apoptosis by Flow cytometry; **(C)** the protein levels of ABCA1 by Immunoblotting. Next, INS-1 cells were cultured in a medium containing 0 or 5 mM cholesterol in the presence or absence of 10 nM Exendin-4 (EX-4) for 24 h and examined for **(D)** cell viability by MTT assay; **(E)** cell apoptosis by Flow cytometry; **(F)** insulin secretion by insulin ELISA kit; **(G)** the protein levels of ABCA1 by Immunoblotting; **(H)** intracellular cholesterol concentration using cholesterol assay kits; **(I)** intracellular lipid deposition by Oil Red O and BODIPY staining. **(J)** The cholesterol efflux from INS-1 cells by BODIPY-cholesterol assay; *n* = 3 for each experiment. *P < 0.05, **P < 0.01, ***P < 0.005, compared with control group, ^#^*P* < 0.05, ^##^*P* < 0.01, compared with CHO + EX-4 group.

In the present study, the effects of GLP-1 on ABCA1 expression and cholesterol accumulation-induced toxicity in INS-1 cells were first verified. INS-1 cells were cultured in different concentrations of cholesterol (0, 1, 2.5, 5, or 10 mM) for 24 h. As depicted in Figures 1A–C, cholesterol stimulation inhibited the cell viability, promoted cell apoptosis, and decreased the protein levels of ABCA1 in a concentration-dependent manner, indicating the occurrence of cholesterol-induced toxicity in INS-1 cells.

Next, INS-1 cells were cultured in a medium containing 5 mM cholesterol in the presence or absence of 10 nM Exendin-4 (EX-4; GLP-1 agonist) for 24 h. Cholesterol could cause suppression of cell viability (Figure 1D) and induction in apoptosis (Figure 1E). The single treatment of EX-4 treated did not significantly affect the cell viability and apoptosis but reversed cholesterol-induced cell viability inhibition and apoptosis (Figures 1D,E). The insulin secretion and the protein levels of ABCA1 were reduced by cholesterol and increased by EX-4 (Figures 1F,G). Moreover, cholesterol stimulation significantly increased the intracellular cholesterol concentration. While EX-4 treatment decreased the intracellular cholesterol content and also reduced cholesterol-induced increase in intracellular cholesterol content (Figure 1H); the Oil Red O and BODPIY staining showed that in cholesterol-stimulated INS-1 cells, lipid droplet accumulation was markedly increased compared to that in the control group while it decreased after EX-4 treatment alone or cholesterol co-treatment compared with that in the single cholesterol group (Figure 1I). Meanwhile, the BODIPY-cholesterol efflux assay indicated that EX-4 treatment alone or cholesterol co-treatment dramatically increased cholesterol efflux from INS-1 cells compared with the single cholesterol stimulation group or the control group (Figure 1J). All the results indicated that GLP-1 agonist EX-4 improves cholesterol accumulation-induced toxicity in INS-1 cells by increasing the cholesterol efflux from INS-1 cells.

### AMPK Signaling Is Involved in GLP-1 Regulation of ABCA1 Expression and Cholesterol-Induced Toxicity

**FIGURE 2:**
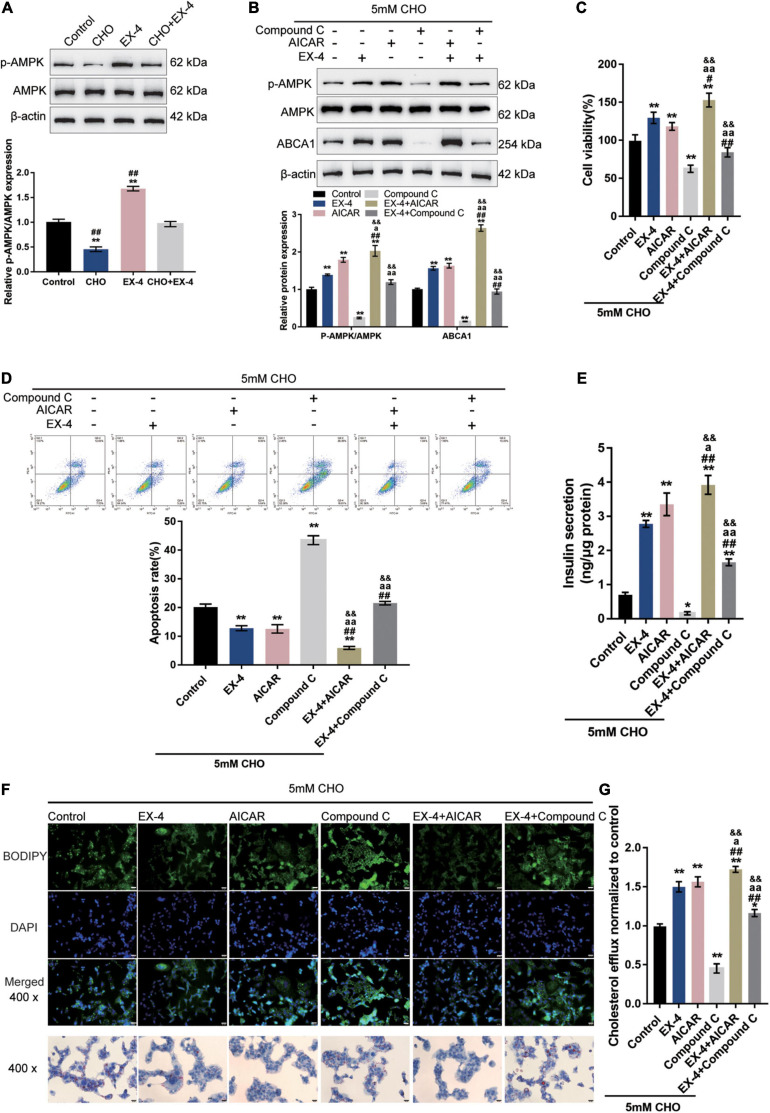
AMPK signaling is involved in GLP-1 regulation of ABCA1 expression and cholesterol-induced toxicity. **(A)** INS-1 cells were cultured in a medium containing 0 and 5 mM cholesterol in the presence or absence of 10 nM EX-4 for 24 h and examined for the protein levels of p-AMPK and AMPK by immunoblotting. Next, INS-1 cells were treated with EX-4 in the presence of AMPK activator AICAR or AMPK inhibitor Compound C under the stimulation of cholesterol and examined for **(B)** the protein levels of ABCA1 by immunoblotting; **(C)** cell viability by MTT assay; **(D)** cell apoptosis by Flow cytometry; **(E)** insulin secretion by insulin ELISA kit; **(F)** intracellular lipid deposition by Oil Red O and BODIPY staining. **(G)** The cholesterol efflux from INS-1 cells by BODIPY-cholesterol assay; *n* = 3 for each experiment. *P < 0.05, **P < 0.01, compared with the control group, ^#^*P* < 0.05, ^##^*P* < 0.01 compared with CHO + EX-4 group, ^a^*P* < 0.05, ^aa^*P* < 0.01 compared with CHO + AICAR group, ^&&^*P* < 0.01 compared with CHO + Compound C group.

The potential involvement of AMPK signaling in GLP-1 protection of INS-1 cells against cholesterol-induced toxicity was investigated. As depicted in Figure 2A, cholesterol stimulation significantly decreased, while EX-4 treatment increased the phosphorylation of AMPK and could reverse the effect of cholesterol stimulation in INS-1 cells, suggesting the involvement of AMPK signaling. Next, INS-1 cells under cholesterol stimulation were treated with EX-4, AMPK activator AICAR or AMPK inhibitor Compound C and examined for related indexes. Under cholesterol stimulation, EX-4 and AICAR increased the ratio of p-AMPK/AMPK, ABCA1 protein levels, and cell viability, which were further promoted by their co-treatment (Figures 2B,C). In contrast, Compound C reduced the ratio of p-AMPK/AMPK, ABCA1 protein levels, and cell viability, which could be reversed by EX-4 co-treatment (Figures 2B,C). Consistently, under cholesterol stimulation, EX-4 and AICAR reduced the cell apoptosis, which was further promoted by their co-treatment (Figure 2D). Contrastingly, Compound C increased cell apoptosis, which could be reversed by EX-4 co-treatment (Figure 2D). EX-4 or AICAR treatment dramatically increased insulin secretion and was further increased by co-treatment of them (Figure 2E). By contrast, Compound C decreased insulin secretion, which could be reversed by EX-4 co-treatment (Figure 2E). As for the lipid droplet accumulation, cholesterol stimulation-induced lipid droplet accumulation was attenuated by EX-4 or AICAR and was further attenuated by co-treatment of them (Figure 2F). Compound C increased lipid droplet accumulation, which could be reversed by EX-4 co-treatment (Figure 2F). Regarding the intracellular cholesterol homeostasis, cholesterol efflux was increased by EX-4 and AICAR and was further increased by their co-treatment (Figure 2G). In contrast, Compound C decreased cholesterol efflux, which could be reversed by EX-4 co-treatment (Figure 2G). The above findings indicate that AMPK activation enhances GLP-1 protection of INS-1 cells against cholesterol-induced toxicity.

### GLP-1 Regulates ABCA1 Expression Through AMPK-Dependent Inhibition on PARP1 Activity

**FIGURE 3:**
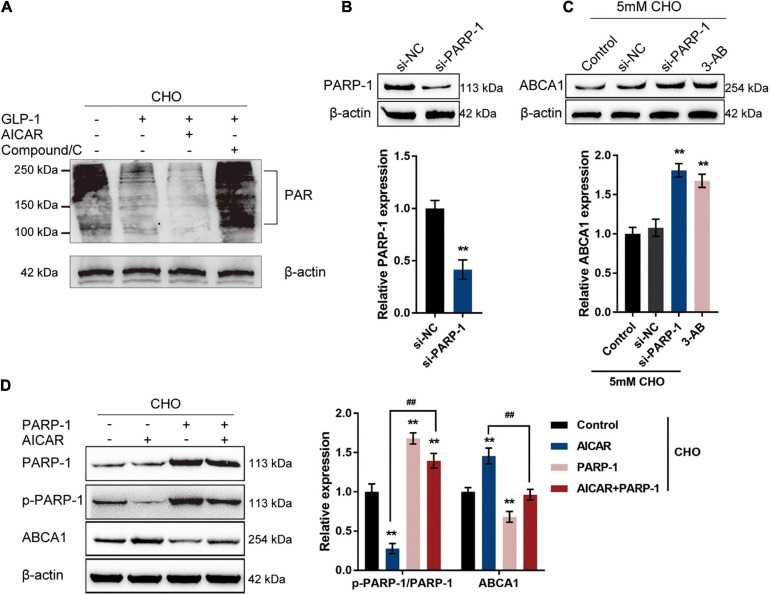
GLP-1 regulates ABCA1 expression through AMPK-dependent inhibition on PARP1 activity. **(A)** Cholesterol-stimulated INS-1 cells were pre-treated with AMPK activator AICAR or AMPK inhibitor Compound C for 4 h before the addition of GLP-1 and incubated for another 24 h. The protein levels of PAR in INS-1 cell lysates were determined by immunoblotting. **(B)** PARP-1 knockdown was generated in INS-1 cells by the transfection of si-PARP-1. The transfection efficiency was confirmed by immunoblotting. **(C)** Cholesterol-stimulated INS-1 cells were transfected with si-PARP-1 or treated with PARP-1 inhibitor 3-AB and examined for the protein levels of ABCA1 by immunoblotting. **(D)** Cholesterol-stimulated INS-1 cells were treated with AMPK activator AICAR or PARP-1 alone or co-treated with AICAR and PARP-1 and examined for the protein levels of p-PARP-1 and ABCA1 by immunoblotting. **P < 0.01 compared with si-NC group or control group, ^##^*P* < 0.01 compared with AICAR group.

Through the use of NAD+ as a substrate, a PARylation process takes place, during which PARP1 synthesizes PAR, cellular NAD+ and ATP are depleted, and transcription factors such as NF-κB are activated (Oliver et al., 1999; Andreone et al., 2003; Rouleau et al., 2010; Bai et al., 2011); therefore, the protein level of PAR could be considered as an indication of PARP1 activity. The specific effects of AMPK phosphorylation on PARP1 activity and ABCA1 expression were subsequently. Cholesterol-stimulated INS-1 cells were cultured with GLP-1 with the pre-treatment of AMPK activator AICAR or AMPK inhibitor Compound C to examine whether AMPK activation could repress PARP1 activity and consequent PARylation. With GLP-1 treatment, a cholesterol-induced increase in the protein level of PAR was decreased in INS-1 cells compared with that in a single cholesterol stimulation group (Figure 3A). Pre-treatment with AICAR was significantly enhanced, while pre-treatment with Compound C attenuated the suppressive effects of GLP-1 on the PAR protein level (Figure 3A). These findings suggest that AMPK activation enhances GLP-1-caused suppression on PARP-1 activity upon cholesterol stimulation.

To further confirm the involvement of AMPK-dependent inhibition on PARP-1 activity in the GLP-1 regulation of ABCA1 expression, PARP-1 knockdown was generated in INS-1 cells by the transfection of si-PARP-1. The transfection efficiency was confirmed by immunoblotting (Figure 3B). Cholesterol-stimulated INS-1 cells were transfected with si-PARP-1 or treated with PARP-1 inhibitor 3-AB, and the ABCA1 protein level was examined; as illustrated in Figure 3C, the protein levels of ABCA1 were significantly increased by PARP-1 knockdown or PARP-1 inhibitor 3-AB, compared with those in the control or si-NC (negative control) group. Next, cholesterol-stimulated INS-1 cells were treated with AMPK activator AICAR or PARP-1 overexpression alone or co-treated with AICAR and PARP-1 and examined for the protein levels of p-PARP-1 and ABCA1. As depicted in Figure 3D, AICAR treatment significantly inhibited the phosphorylation of PARP-1 while it increased the ABCA1 protein level; contrastingly, PARP-1 treatment promoted PARP-1 phosphorylation while it decreased ABCA1 protein level upon cholesterol stimulation. More importantly, although AICAR acts on PARP-1 phosphorylation and ABCA1 protein level in the presence or absence of PARP1 treatment, the effects of AICAR could be partially attenuated by PARP-1 treatment. These data indicate that GLP-1 regulation of ABCA1 protein levels involves AMPK-dependent inhibition on PARP-1 phosphorylation and PAPR-1 activity.

### AMPK Inhibits PARP1 Activity to Attenuate Cholesterol-Induced Toxicity

**FIGURE 4:**
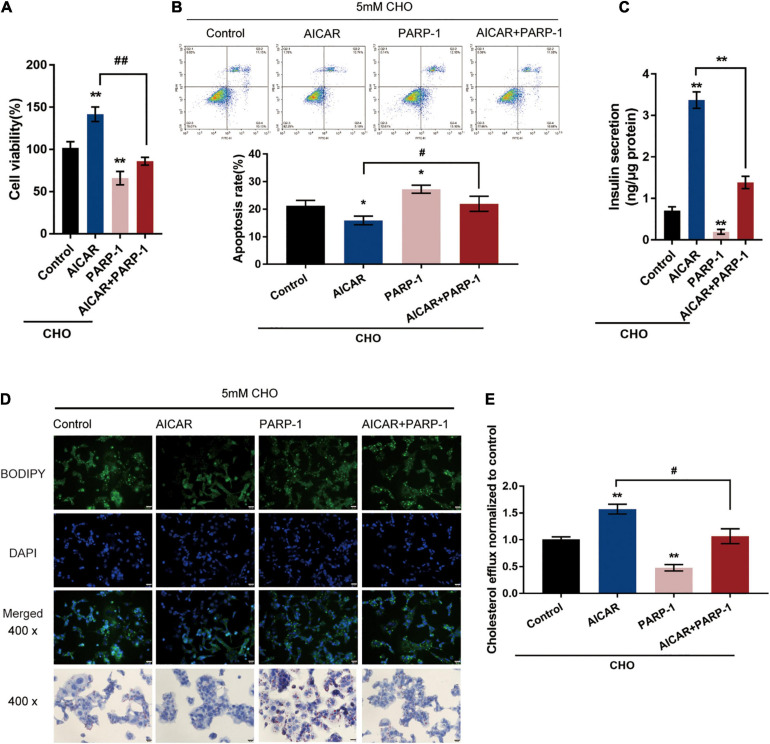
AMPK inhibits PARP1 activity to attenuate cholesterol-induced toxicity. Cholesterol-stimulated INS-1 cells were treated with AICAR or PARP-1 alone or co-treated with AICAR and PARP-1 and examined for **(A)** cell viability by MTT assay; **(B)** cell apoptosis by Flow cytometry; **(C)** insulin secretion by insulin ELISA kit; **(D)** intracellular lipid deposition by Oil Red O and BODIPY staining. **(E)** The cholesterol efflux from INS-1 cells by BODIPY-cholesterol assay. *P < 0.05, **P < 0.01 compared with control group, ^#^*P* < 0.05, ^##^*P* < 0.01 compared with AICAR group.

The potential attenuation of cholesterol-induced toxicity through PARP-1 activity by AMPK activation was further investigated. Cholesterol-stimulated INS-1 cells were treated with AICAR or PARP-1 alone or co-treated with AICAR and PARP-1 and examined for related indexes. AICAR significantly promoted cell viability and inhibited the cell apoptosis in INS-1 cells, while PARP-1 exerted opposite effects; when PARP-1 was overexpressed, the effects of AICAR on INS-1 cells were partially attenuated (Figures 4A,B). As for insulin secretion, AICAR was notably promoted, while PARP-1 markedly restrained insulin secretion; the effects of AICAR on insulin secretion were reversed by PARP-1 (Figure 4C). Consistently, AICAR was significantly reduced the intracellular lipid deposition, while PARP-1 exerted an opposite effect; the effects of AICAR on the intracellular lipid deposition were reversed by PARP-1 (Figure 4D). Regarding cholesterol homeostasis, AICAR significantly increased, while PARP-1 inhibited the cholesterol efflux from INS-1 cells (Figure 4E); similarly, the effects of AICAR on cholesterol efflux from INS-1 cells were reversed by PARP-1 (Figure 4E). These data indicate that AMPK activation could attenuate cholesterol-induced toxicity; the beneficial effects of AMPK activation are attenuated when PARP-1 is activated.

### PAPR-1 Inhibits LXR-Induced ABCA1 Transcription in INS-1 Cells

**FIGURE 5:**
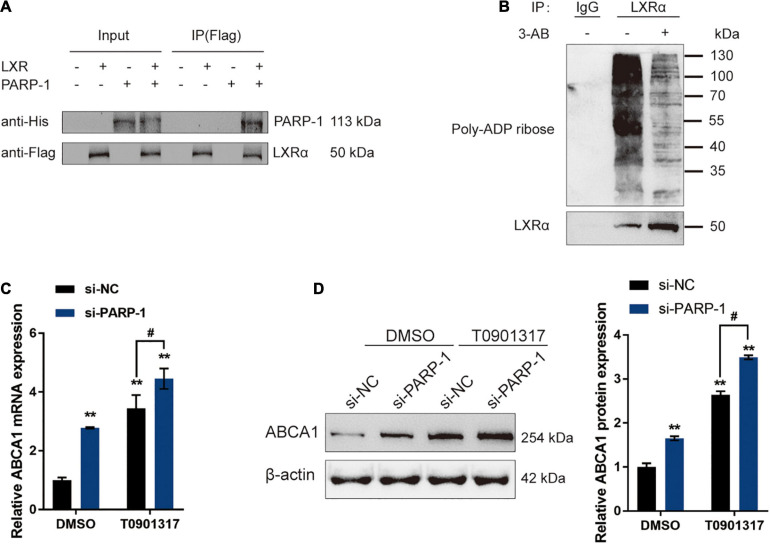
PARP-1 inhibits LXR-induced ABCA1 transcription in INS-1 cells. **(A)** Confirmation of the interaction between LXR and PARP-1 in INS-1 cells using coimmunoprecipitation (Co-IP) assay. **(B)** LXRα poly(ADP-ribosyl)ation was detected in INS-1 by Poly(ADP-ribosyl)ation Assay. **(C,D)** INS-1 cells were transfected with si-PARP-1 in the presence or absence of LXR receptor agonist T0901317 and examined for the mRNA expression and protein levels of ABCA1 by real-time PCR and immunoblotting. *n* = 3 for each experiment. **P < 0.01 compared with the si-NC + DMSO group, ^#^*P* < 0.05 compared with the si-NC + T0901317 group.

The potential inhibition of LXR-induced ABCA1 expression by PARP-1 in INS-1 cells was investigated. In Co-IP assays, Flag-LXR and His-PARP-1 vectors were constructed and co-transfected into INS-1 cells. The results from immunoblotting showed that PARP-1 protein could interact with the LXR protein in INS-1 cells (Figure 5A). LXRα poly(ADP-ribosyl)ation could also be detected in INS-1, and the poly(ADP-ribosyl)ation of LXRα was reduced upon inhibition of PARP-1 by 3-AB (Figure 5B). Moreover, INS-1 cells were transfected with si-PARP-1 in the presence or absence of LXR receptor agonist T0901317, and the expression and protein levels of ABCA1 were examined. The LXR receptor agonist T0901317 treatment dramatically promoted the expression and protein levels of ABCA1, which could be further boosted by PARP-1 knockdown, but even without LXR receptor agonist T0901317 treatment, PARP-1 knockdown alone also caused an increase in ABCA1 expression and protein level (Figures 5C,D). These data indicate that PAPR-1 induces LXRα poly(ADP-ribosyl)ation, therefore inhibiting LXR-induced ABCA1 expression in INS-1 cells.

### GLP-1 Inhibits PARP-1 to Improve Islet Function in T2DM Rats

**FIGURE 6:**
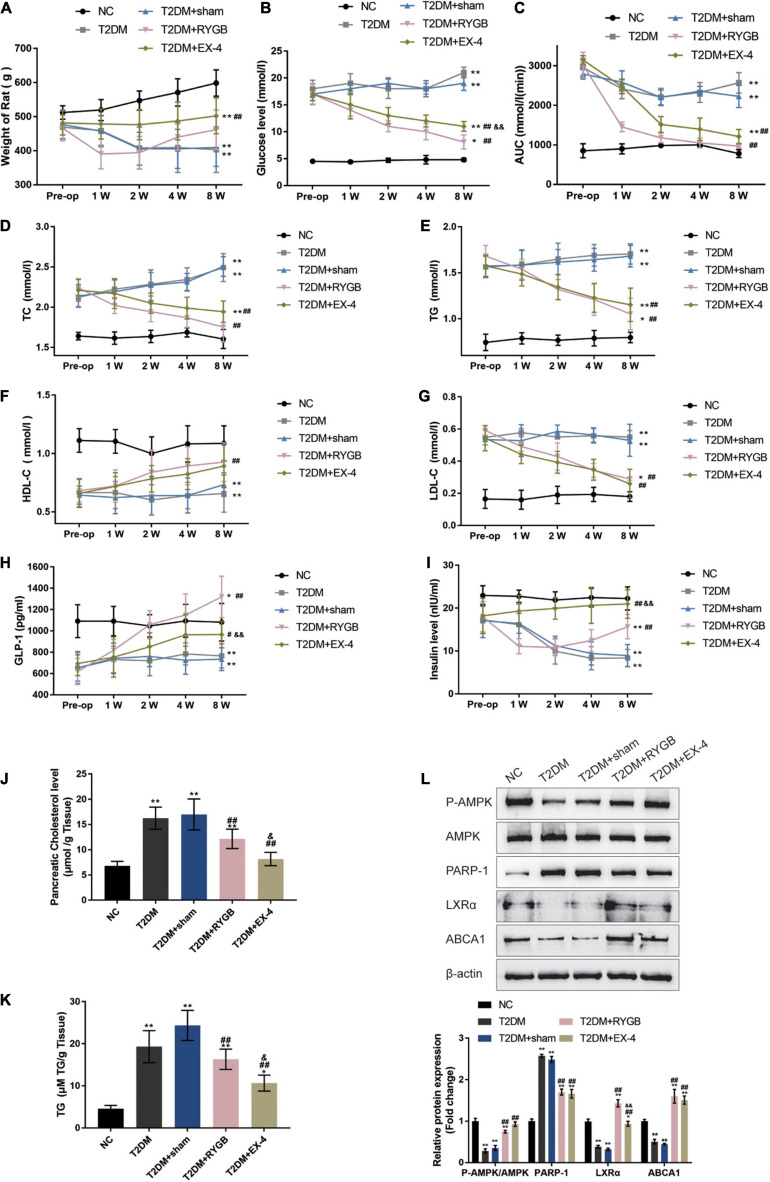
GLP-1 inhibits PARP-1 to improve islet function in T2DM rats. Sprague-Dawley (SD) rats were randomly assigned into five groups: non-treatment control group (*n* = 6), streptozotocin (STZ)-induced T2DM group (*n* = 6), T2DM plus sham surgery group (*n* = 7), T2DM plus RYGB surgery group (*n* = 9), and T2DM plus EX-4 treatment group (*n* = 8). At pre-operation (RYGB surgery or EX-4 injection), 1, 2, 4, and 8 weeks, the body weight **(A)**, fasting blood glucose levels (FBG) in serum **(B)**, the area under the curve (AUC) in serum for the OGTT (oral glucose tolerance test, mmol/L/hour) **(C)**, total cholesterol (TC) in serum **(D)**, triglyceride (TG) in serum **(E)**, low-density lipoprotein cholesterol (LDL-C) in serum **(F)**, and high-density lipoprotein cholesterol (HDL-C) in serum **(G)**, GLP-1 levels in serum **(H)**, and insulin levels in serum **(I)** were examined. **(J,K)** The TC and TG levels in pancreatic tissues of rats were determined. **(L)** The protein levels of p-AMPK, AMPK, PARP-1, LXRα, and ABCA1 were examined by immunoblotting in rats’ pancreatic tissues. *n* = 6, *P < 0.05, **P < 0.01 compared with NC group, ^#^*P* < 0.05, ^##^*P* < 0.01 compared with T2DM + sham group, ^&^*P* < 0.05, ^&&^*P* < 0.01 compared with T2DM + RYGB group.

To further confirm these *in vitro* findings, a T2DM model in Sprague-Dawley (SD) rats was established, and functional analyses were performed. SD rats were randomly assigned into five groups: the non-treatment control group, the streptozotocin (STZ)-induced T2DM group, the STZ-induced T2DM plus sham surgery group, the STZ-induced T2DM plus RYGB surgery group, and the STZ-induced T2DM plus EX-4 treatment group. At pre-operation (RYGB surgery or EX-4 injection), at weeks 1, 2, 4, and 8, related indexes were monitored. One week following surgery, significant weight loss was observed T2DM + RYGB group; from weeks 2 to 4, rats in all the T2DM groups had lower body weight than those in the normal group, among which the body weight of rats in the T2DM + RYGB group increased slowly post-operation, slightly higher than that in T2DM and T2DM + sham. Contrastingly, the body weight of rats in the T2DM + EX-4 group was stable and increased slowly (Figure 6A). FBG levels in rat serum in T2DM + RYGB and T2DM + EX-4 groups decreased significantly after surgery, while those in T2DM and T2DM + sham groups increased steadily (Figure 6B). The AUC in serum of rats of the T2DM + RYGB and T2DM + EX-4 group significantly decreased after surgery, while the AUC of T2DM and T2DM + sham group slightly decreased (Figure 6C). The TC (Figure 6D) and TG (Figure 6E) contents in serum of rats in T2DM + RYGB and T2DM + EX-4 groups significantly reduced after surgery, while those in T2DM and T2DM + sham groups increased steadily. The HDL-C in serum of rats significantly increased in T2DM + RYGB and T2DM + EX-4 groups after surgery while remained constant in T2DM and T2DM + sham groups (Figure 6F). The LDL-C in serum of rats in T2DM and T2DM + sham groups was significantly higher than the NC group, while LDL-C decreased in T2DM + RYGB and T2DM + EX-4 groups after surgery when compared with the T2DM group (Figure 6G). Regarding the serum GLP-1 and insulin levels, the levels of GLP-1 in T2DM and T2DM + sham group were significantly lower than NC group, compared with the T2DM + sham group, GLP-1 levels increased dramatically in T2DM + RYGB and T2DM + EX-4 groups (Figure 6H); insulin content was decreased in T2DM and T2DM + sham groups when compared with NC group. While insulin content was first reduced and then gradually increased in the T2DM + RYGB group, it risen continuously in the T2DM + EX-4 group compared with the T2DM + sham group (Figure 6I). Besides, the cholesterol and TG levels in pancreatic tissues of rats were also determined at the end of the EX-4 or RYGB treatment. The level of pancreatic cholesterol in the T2DM and T2DM + sham group was markedly higher than the NC group, while compared to the T2DM + sham group, pancreatic cholesterol level dramatically decreased in T2DM + RYGB and T2DM + EX-4 groups (Figure 6J). When compared to the control group, TG contents in pancreatic tissue of rats in T2DM and T2DM + sham groups significantly increased, while it observably decreased after surgery in T2DM + RYGB and T2DM + EX-4 groups (Figure 6K). These data all indicate that T2DM rats showed significant improvement in glucose metabolism and lipid metabolism after RYGB surgery or EX-4 treatment.

At the end of the EX-4 or RYGB treatment, the protein levels of p-AMPK, AMPK, PARP-1, LXRα, and ABCA1 were examined in rats’ pancreatic tissues. p-AMPK/AMPK, LXRα, and ABCA1 protein levels were decreased, and PARP-1 was increased in T2DM and T2DM + sham groups when compared with the NC control group; on the contrary, p-AMPK/AMPK, LXRα, and ABCA1 protein levels were increased, and PARP-1 was decreased in T2DM + RYGB and T2DM + EX-4 groups when compared with T2DM group (Figure 6L). These findings are consistent with the *in vitro* results that GLP-1 induces AMPK phosphorylation, inhibits PARP-1, therefore increasing LXRα-mediated ABCA1 expression.

**FIGURE 7:**
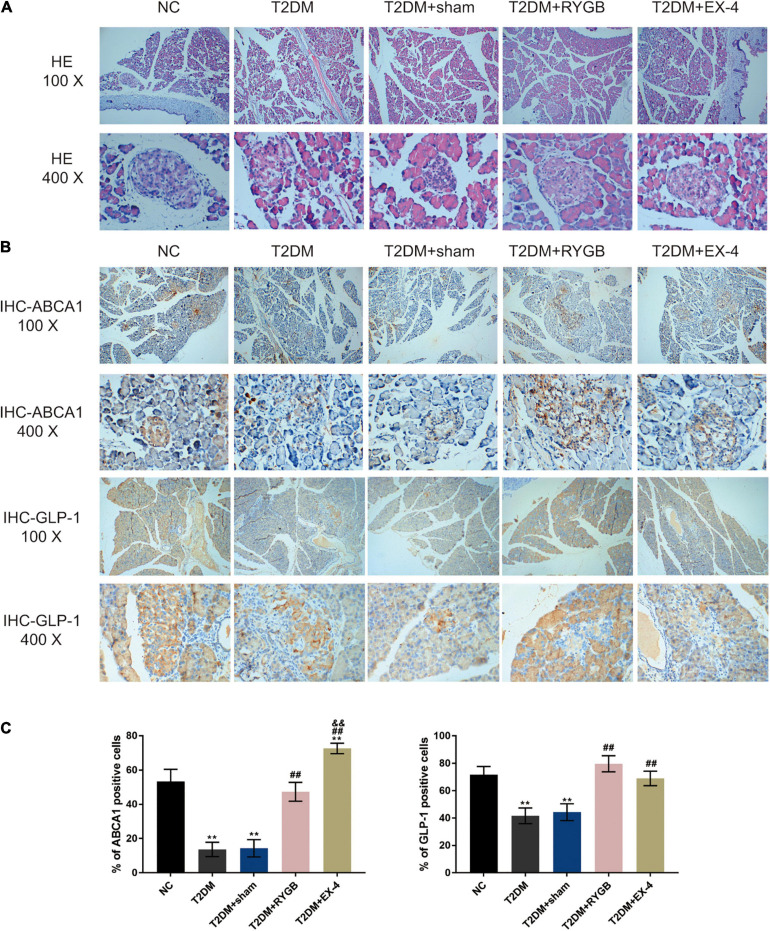
The pancreatic islet morphology and ABCA1 and GLP-1 protein levels. Sprague-Dawley (SD) rats were randomly assigned into five groups: non-treatment control group (*n* = 6), streptozotocin (STZ)-induced T2DM group (*n* = 6), T2DM plus sham surgery group (*n* = 7), T2DM plus RYGB surgery group (*n* = 9), and T2DM plus EX-4 treatment group (*n* = 8). **(A)** The histopathology of rats’ pancreatic tissues was examined by H&E staining. *n* = 6. **(B,C)** The protein contents and distribution of ABCA1 and PARP-1 were examined by IHC staining in rats’ pancreatic tissues. *n* = 6, *P < 0.05, **P < 0.01 compared with NC group, ^##^*P* < 0.01 compared with T2DM + sham group, ^&&^*P* < 0.01 compared with T2DM + RYGB group.

To confirm the pancreatic islet morphology, the pancreatic islets were observed by H&E staining under a microscope. As illustrated in Figure 7A, the size of islets in the T2DM group and T2DM + sham group was significantly reduced, the vacuole area increased, the boundary between the islets and the surrounding area was not clear, and the arrangement was loose. While the size of the islets was larger, the arrangement was more regular, and the boundary was more evident between the islet and the acinus in NC, T2DM + RYGB, and T2DM + EX-4 groups (Figure 7A). IHC staining also demonstrated that the protein contents of ABCA1 and GLP-1 decreased in T2DM and T2DM + sham groups while it increased in T2DM + RYGB and T2DM + EX-4 groups (Figures 7B,C).

## Discussion

In this study, a high concentration cholesterol-induced lipotoxicity was observed in INS-1 cells, including inhibited cell viability, enhanced cell apoptosis, and inhibited cholesterol efflux from INS-1 cells; meanwhile, the ABCA1 protein level was decreased by cholesterol stimulation. Cholesterol-induced toxicity and ABCA1 downregulation were attenuated by GLP-1R agonist EX-4. GLP-1 induced AMPK phosphorylation during the protection against cholesterol-induced toxicity. Under cholesterol stimulation, GLP-1-induced AMPK activation inhibited PARP-1 activity, thereby attenuating cholesterol-induced toxicity in INS-1 cells. In INS-1 cells, PARP-1 directly interacted with LXR, leading to the poly(ADP-ribosyl)ation of LXRα and downregulation of LXR-mediated ABCA1 expression. In the STZ-induced T2DM model in rats, RYGB surgery or EX-4 treatment improved the glucose metabolism and lipid metabolism in rats through GLP-1 inhibition of PARP-1 activity.

GLP-1 can stimulate islet β cells to secrete insulin and inhibit glucagon secretion (Kreymann et al., 1987; Buchwald et al., 2004; Holst, 2004). Moreover, GLP-1 can reduce the islet β cell apoptosis rate and stimulate β cell proliferation and regeneration (Xu et al., 1999; Kwon et al., 2009). Lipotoxicity is the main feature of T2DM, but the protective effect of GLP-1 regulation of cholesterol efflux from pancreatic β-cells against lipotoxicity has not been reported as of yet. The deleterious effects of free fatty acids (FFAs) on glucose homeostasis are commonly referred to as lipotoxicity (Opazo-Rios et al., 2020). A substantial body of data proves that increased FFAs induce insulin resistance and pancreatic β-cell dysfunction, entailing the two significant defects underlying T2DM pathophysiology (Lytrivi et al., 2020). Numerous studies have shown that high concentrations of FFA are noxious to β-cells, and when infused in large amounts in humans and rodents, they produce suppression of insulin secretion (Hauke et al., 2018; Weir, 2020). Therefore, reducing saturated FFA-mediated cell toxicity may serve as a novel treatment regimen for refractory diabetic nephropathy. However, it has been challenging to find clear correlations between β-cell dysfunction and FFA, and no drug and strategy aimed at ameliorating saturated FFA-mediated cell toxicity have been developed in clinical settings (Kume and Maegawa, 2020). In this study, it was found that high-fat content can inhibit pancreatic β-cell cholesterol efflux and down-regulate the expression of ABCA1 in islet INS-1 cells. Simultaneously, GLP-1 treatment upregulates the expression of ABCA1, thereby repairing the cholesterol efflux from islet β-cells that have sustained damage by lipotoxicity, reducing the cellular cholesterol content, improving cellular lipid deposition, and restoring islet β-cell function, which is consistent with the results of previous studies (Murao et al., 2009; Miyai et al., 2011). Cholesterol efflux from pancreatic islet β cells mainly occurs through ABCA1, which expels cholesterol out of the cells through the cell membrane to combine with the cholesterol acceptor Apolipoprotein A-I (ApoA-I) (Luu et al., 2013). ABCA1 knockout mice had an impaired glucose tolerance but normal insulin sensitivity, suggesting that these mice suffered from islet β-cell dysfunction; meanwhile, ABCA1 knockout mice had low total plasma cholesterol levels (Oram and Lawn, 2001). These findings all indicate that GLP-1-induced ABCA1 upregulation contributes to the cholesterol homeostasis in pancreatic β-cells and protects pancreatic β-cells from cholesterol-induced toxicity.

Cholesterol efflux has long been regarded as a critical mechanism of maintaining intracellular cholesterol homeostasis, which could be primarily regulated by the LXR transcription factors and their targeted genes, the ATP-binding cassette (ABC) cholesterol transporters ABCA1 and ABCG1 (Robichaud and Ouimet, 2019). In hepatocytes, GLP-1 inhibits intracellular lipid production through the cAMP/AMPK signaling pathway, and this pathway can act directly upon LXR (Henquin, 2000). In pancreatic β cells, GLP-1 binds GLP-1R to promote the conversion of ATP into cytoplasmic cAMP, further activating the intracellular PKA/AMPK signaling pathway and promoting glucose-stimulated insulin secretion (GSIS) (Kahn, 2001). These pieces of evidence suggest that after GLP-1 binds to GLP-1R on the surface of islet β cells, it is likely to activate the AMPK signaling pathway to act on the nucleus LXRα and promote the activation of cell membrane ABCA1, and thus participate in the process of cholesterol efflux from islet β cells. In the present study, cholesterol-induced suppression on AMPK phosphorylation and ABCA1 protein expression could indeed be attenuated by GLP-1R agonist EX-4 and further abolished by AMPK activator AICAR while it is enhanced by AMPK inhibitor Compound C. Consistently, AMPK activator AICAR was attenuated, while AMPK inhibitor Compound C aggravated cholesterol-induced toxicity. These data indicate that GLP-1 protects islet β cells against cholesterol-induced toxicity through the activation of AMPK, and LXR might be involved.

Inflammation has been implicated in the pathophysiology of type 2 diabetes (Eguchi and Nagai, 2017). In this study, the late cell apoptosis in INS-1 cells was markedly increased when exposed to cholesterol; moreover, cholesterol inhibited the phosphorylation of AMPK, which was involved in the inflammatory response. The mechanisms of inflammation and β-cell death have been subject to numerous studies. During insulitis, activated macrophages and T-cells release cytokines, including interleukin (IL) 1β, tumor necrosis factor (TNF)-α and interferon (IFN)-γ, close to β-cells, contributing to β-cell dysfunction and death (Berchtold et al., 2016; Boni-Schnetzler and Meier, 2019). Moreover, previous findings suggest that islet β-cells auto-secrete cytokines, which induce inflammatory responses (Allagnat et al., 2012). These results indicate that cholesterol decreased in proliferation and increased apoptosis of INS-1 cells, which could potentially be involved in the cytokine secretion and inflammatory response and is noxious to cell survival.

As mentioned previously, PARP1 synthesizes PAR for “PARylation” of both itself and other proteins (nuclear and cytoplasmic) using NAD+ and ATP, leading to the activation of transcription factors such as NF-κB and AP-1 (Oliver et al., 1999; Andreone et al., 2003; Rouleau et al., 2010; Bai et al., 2011). Moreover, PARP-1 also participates in lipid homeostasis (Vida et al., 2017). In this study, it was first demonstrated that in islet β cells, GPL-1-induced AMPK activation suppressed PARP-1 activity to increase ABCA1 protein expression. In contrast, PARP-1 over-activation attenuated the promotive effects of GPL-1-induced AMPK activation on ABCA1 protein expression. Consistently, AMPK activator AICAR was attenuated, while PARP-1 over-activation aggravated cholesterol-induced toxicity in INS-1 cells, indicating that in islet β cells, PARP-1 also regulates cholesterol efflux through AMPK activation and ABCA1 protein, therefore affecting the GLP-1 protection against cholesterol-induced toxicity.

A previous study has revealed that PARP-1 could inhibit LXR-induced ABCA1 expression and cholesterol efflux from macrophages (Shrestha et al., 2016). In this study, the interaction between PARP-1 and LXR in islet β cells was demonstrated. PARP-1-induced LXRα poly(ADP-ribosyl)ation could be repressed by PARP-1 inhibitor 3-AB. PARP-1 knockdown increased, while LXR agonist T0901317 further increased ABCA1 protein levels. These data indicate that in islet β cells, PARP-1 could also inhibit LXR-mediated ABCA1 expression by interacting with LXR to induce LXRα poly(ADP-ribosyl)ation.

Bariatric surgery can significantly alleviate T2DM and usually can substantially improve blood glucose levels prior to weight loss after surgery (Madsbad et al., 2014). Peterli et al. (2009) found that GLP-1 levels after meals were increased dramatically in patients 2 months after SG surgery. Another group reported that GLP-1 was elevated in the postprandial period in diabetic patients 6 weeks after SG surgery and persisted for at least 1 year (Papamargaritis et al., 2013). In this study, the STZ-induced T2DM model in SD rats was established. It was observed that in T2DM rats, the expression of ABCA1 in the islet tissues was significantly weakened, the cholesterol efflux was blocked, and the cholesterol content in the tissues increased considerably. On the contrary, RYGB surgery or EX-4 treatment improved glucose metabolism and lipid metabolism. Meanwhile, in T2DM rats who underwent RYGB surgery or EX-4 treatment, the expression of GLP-1, ABCA1, and LXRβ was increased, the cholesterol content was decreased, the phosphorylation of AMPK was enhanced. In contrast, the protein level of PARP-1 was reduced compared with the T2DM or T2DM + sham groups. The results indicate that the GLP-1 upregulation post-surgery can upregulate the expression of ABCA1 protein, promote the cholesterol efflux from islet β cells, reduce pancreatic cholesterol content, thereby improving the glucose metabolism and lipid metabolism in T2DM rats. Some previous studies have shown that EX-4 can increase the level of GLP-1 in different body parts. In the research conducted by Candeias et al. (2018), Ex-4 subcutaneous injection enhanced their brain cortical GLP-1 and IGF-1 levels in T2D GK rats. Sahraoui et al. (2015) found that active GLP-1 levels in diabetic mice transplanted with islet grafts were significantly increased following treatment with EX-4 compared to untreated control. They found that low dose EX-4 treatment may protect the β-cells from apoptosis, consequently increased the number of the β-cells. Therefore, in this study, it is believed that EX-4 might also affect the β-cell viability, which might contribute to the increase of serum GLP-1.

## Conclusion

**FIGURE 8:**
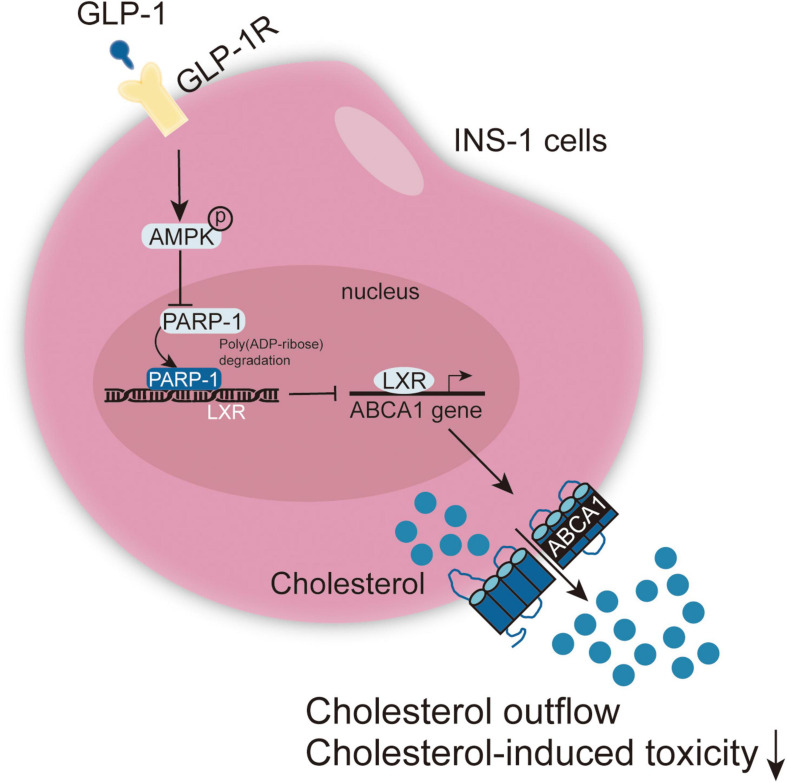
A schematic graph of the mechanism. GLP-1 induces AMPK and LXR-mediated ABCA1 expression and inhibits PARP-1 to protect islet β cell function against cholesterol-induced toxicity *in vitro* and *in vivo* through enhancing cholesterol efflux.

GLP-1 inhibits PARP-1 to protect islet β cell function against cholesterol-induced toxicity *in vitro* and *in vivo* through the enhancement of cholesterol efflux. GLP-1-induced AMPK and LXR-mediated ABCA1 expression are involved in GLP-1 protective effects (Figure 8).

## Data Availability Statement

The raw data supporting the conclusions of this article will be made available by the authors, without undue reservation, to any qualified researcher.

## Ethics Statement

The animal study was reviewed and approved by the Ethics Committee of Third Xiangya Hospital, Central South University.

## Author Contributions

RL, XS, and LYZ contributed to experimental design and supervising the whole experimental process. PL and WL were involved in the experimental conducting. RL, XS, and LZ contributed to the data analysis and manuscript preparation. LYZ and SZ revised the work critically for important intellectual content. LYZ collected grants. All authors read, revised, and approved the final manuscript.

## Conflict of Interest

The authors declare that the research was conducted in the absence of any commercial or financial relationships that could be construed as a potential conflict of interest. The reviewer JY declared a shared affiliation with one of the authors LZ to the handling Editor at time of review.

## Abbreviations

ABC: ATP-binding cassette
ABCA1: ATP-binding cassette transporter A1
GLP-1: glucagon-like peptide-1
GSIS: glucose-stimulated insulin secretion
HDL: high-density lipoprotein
HDL-C: high-density lipoprotein cholesterol
H&E: Hematoxylin and eosin
IHC: immunohistochemical
IP: immunoprecipitation
LDL: low-density lipoprotein
LSG: laparoscopic sleeve gastrectomy
LXR: liver X receptor
OGTT: oral glucose tolerance test
PARP: poly ADP-ribose polymerase
RCT: reverse cholesterol transport
RYGB: Roux-en-Y gastric bypass
STZ: streptozotocin
TC: total cholesterol
TG: total triglyceride
T2DM: type 2 diabetes.
